# Absence of Fluoride Varnish–Related Adverse Events in Caries Prevention Trials in Young Children, United States

**DOI:** 10.5888/pcd14.160372

**Published:** 2017-02-16

**Authors:** Raul I. Garcia, Steven E. Gregorich, Francisco Ramos-Gomez, Patricia A. Braun, Anne Wilson, Judith Albino, Tamanna Tiwari, Maya Harper, Terrence S. Batliner, Margaret Rasmussen, Nancy F. Cheng, William Santo, Paul L. Geltman, Michelle Henshaw, Stuart A. Gansky

**Affiliations:** 1 Center for Research to Evaluate and Eliminate Dental Disparities, Boston University Henry M. Goldman School of Dental Medicine, Boston, Massachusetts; 2 Center to Address Disparities in Children’s Oral Health, School of Dentistry, University of California, San Francisco; 3 Department of Medicine, School of Medicine, University of California, San Francisco; 4 School of Dentistry, University of California, Los Angeles; 5 Center for Native Oral Health Research, Colorado School of Public Health, University of Colorado Anschutz Medical Campus, Aurora, Colorado; 6 School of Dental Medicine, University of Colorado Anschutz Medical Campus, Aurora, Colorado

## Abstract

**Introduction:**

Fluoride varnish is an effective prevention intervention for caries in young children. Its routine use in clinical care is supported by meta-analyses and recommended by clinical guidelines, including the US Preventive Services Task Force (B rating). This report is the first prospective systematic assessment of adverse events related to fluoride varnish treatment in young children.

**Methods:**

We determined the incidence of adverse events related to fluoride varnish treatment in 3 clinical trials on the prevention of early childhood caries, conducted under the auspices of the Early Childhood Caries Collaborating Centers, an initiative sponsored by the National Institute of Dental and Craniofacial Research. Each trial incorporated use of fluoride varnish in its protocol and systematically queried all children’s parents or legal guardians about the occurrence of acute adverse events after each fluoride varnish treatment.

**Results:**

A total of 2,424 community-dwelling, dentate children aged 0 to 5 years were enrolled and followed for up to 3 years. These children received a cumulative total of 10,249 fluoride varnish treatments. On average, each child received 4.2 fluoride varnish treatments. We found zero fluoride varnish–related adverse events.

**Conclusion:**

Fluoride varnish was not associated with treatment-related adverse events in young children. Our findings support its safety as an effective prevention intervention for caries in young children.

**MEDSCAPE CME**

Medscape, LLC is pleased to provide online continuing medical education (CME) for this journal article, allowing clinicians the opportunity to earn CME credit.

This activity has been planned and implemented through the joint providership of Medscape, LLC and *Preventing Chronic Disease*. Medscape, LLC is accredited by the American Nurses Credentialing Center (ANCC), the Accreditation Council for Pharmacy Education (ACPE), and the Accreditation Council for Continuing Medical Education (ACCME), to provide continuing education for the healthcare team.

Medscape, LLC designates this Journal-based CME activity for a maximum of 1.00 ***AMA PRA Category 1 Credit(s)™***. Physicians should claim only the credit commensurate with the extent of their participation in the activity.

All other clinicians completing this activity will be issued a certificate of participation. To participate in this journal CME activity: (1) review the learning objectives and author disclosures; (2) study the education content; (3) take the post-test with a 75% minimum passing score and complete the evaluation at http://www.medscape.org/journal/pcd; (4) view/print certificate.

Release date: February 16, 2017; Expiration date: February 16, 2018

**Learning Objectives**

Upon completion of this activity, participants will be able to:

1. Distinguish findings regarding adverse events of fluoride varnish in young children enrolled in caries prevention trials, based on a prospective systematic assessment
2. Determine the clinical implications of these findings regarding adverse events of fluoride varnish in young children enrolled in caries prevention trials
3. Identify potential concerns regarding the safety of fluoride varnish treatment

**EDITOR**

Ellen Taratus

Editor, *Preventing Chronic Disease*

Disclosure: Ellen Taratus has disclosed no relevant financial relationships.

**CME AUTHOR**

Laurie Barclay, MD

Freelance writer and reviewer, Medscape, LLC

Disclosure: Laurie Barclay, MD, has disclosed the following relevant financial relationships:

Owns stock, stock options, or bonds from: Pfizer

**AUTHORS**

Raul I. Garcia, DMD, MMedSc

Center for Research to Evaluate and Eliminate Dental Disparities (CREEDD), Boston University Henry M. Goldman School of Dental Medicine, Boston, Massachusetts

Disclosure: Raul I. Garcia, DMD, MMedSc, has disclosed no relevant financial relationships.

Steven E. Gregorich, PhD

Center to Address Disparities in Children's Oral Health (CAN DO), School of Dentistry, University of California San Francisco; Department of Medicine, School of Medicine, University of California San Francisco, San Francisco, California

Disclosure: Steven E. Gregorich, PhD, has disclosed no relevant financial relationships.

Francisco Ramos-Gomez, DDS, MS, MPH

School of Dentistry, University of California Los Angeles, Los Angeles, California

Disclosure: Francisco Ramos-Gomez, DDS, MS, MPH, has disclosed no relevant financial relationships.

Patricia A. Braun, MD, MPH

Center for Native Oral Health Research (CNOHR), Colorado School of Public Health, University of Colorado Anschutz Medical Campus, Aurora, Colorado

Disclosure: Patricia A. Braun, MD, MPH, has disclosed no relevant financial relationships.

Anne Wilson, DDS, MS

School of Dental Medicine, University of Colorado Anschutz Medical Campus, Aurora, Colorado

Disclosure: Anne Wilson, DDS, MS, has disclosed no relevant financial relationships.

Judith Albino, PhD

Center for Native Oral Health Research (CNOHR), Colorado School of Public Health, University of Colorado Anschutz Medical Campus, Aurora, Colorado

Disclosure: Judith Albino, PhD, has disclosed no relevant financial relationships.

Tamanna Tiwari, BDS, MDS, MPH

School of Dental Medicine, University of Colorado Anschutz Medical Campus, Aurora, Colorado

Disclosure: Tamanna Tiwari, BDS, MDS, MPH, has disclosed no relevant financial relationships.

Maya Harper

Center for Native Oral Health Research (CNOHR), Colorado School of Public Health, University of Colorado Anschutz Medical Campus, Aurora, Colorado

Disclosure: Maya Harper has disclosed no relevant financial relationships.

Terrence S. Batliner, DDS, MBA

Center for Native Oral Health Research (CNOHR), Colorado School of Public Health and School of Dental Medicine, University of Colorado Anschutz Medical Campus, Aurora, Colorado

Disclosure: Terrence S. Batliner, DDS, MBA, has disclosed no relevant financial relationships.

Margaret Rasmussen, MPH

Center to Address Disparities in Children's Oral Health (CAN DO), School of Dentistry, University of California San Francisco, San Francisco, California

Disclosure: Margaret Rasmussen, MPH, has disclosed no relevant financial relationships.

Nancy F. Cheng, MS, MS

Center to Address Disparities in Children's Oral Health (CAN DO), School of Dentistry, University of California San Francisco, San Francisco, California

Disclosure: Nancy F. Cheng, MS, MS, has disclosed the following relevant financial relationships:

Owns stock, stock options, or bonds from: Johnson & Johnson, Amgen, Waters, Perrigo, Dentsply Sirona

William Santo, BA

Center to Address Disparities in Children's Oral Health (CAN DO), School of Dentistry, University of California San Francisco, San Francisco, California

Disclosure: William Santo, BA, has disclosed no relevant financial relationships.

Paul L. Geltman, MD, MPH

Center for Research to Evaluate and Eliminate Dental Disparities (CREEDD), Boston University Henry M. Goldman School of Dental Medicine, Boston, Massachusetts

Disclosure: Paul L. Geltman, MD, MPH, has disclosed no relevant financial relationships.

Michelle Henshaw, DDS, MPH

Center for Research to Evaluate and Eliminate Dental Disparities (CREEDD), Boston University Henry M. Goldman School of Dental Medicine, Boston, Massachusetts

Disclosure: Michelle Henshaw, DDS, MPH, has disclosed no relevant financial relationships.

Stuart A. Gansky, DrPH

Center to Address Disparities in Children's Oral Health (CAN DO), School of Dentistry, University of California San Francisco, San Francisco, California

Disclosure: Stuart A. Gansky, DrPH, has disclosed no relevant financial relationships.

## Introduction

Dental caries in children is highly prevalent; approximately 37% of US children aged 2 to 8 years had dental caries in primary teeth in 2011–2012, with significant disparities related to race/ethnicity and poverty status (1). Effective caries prevention interventions exist, including periodically applying 5% fluoride varnish to children’s primary teeth at the age of primary tooth eruption (2,3). In 2014, the US Preventive Services Task Force (USPSTF) (3) concluded that evidence supported use of fluoride varnish in all children, from birth through age 5 years, and gave a B evidence grade to its use. Professional associations, including the American Academy of Pediatrics (4) and the American Academy of Pediatric Dentistry (5), also endorse fluoride varnish for caries prevention. Third-party payers, including commercial dental insurers and federal programs (eg, Medicaid, State Children’s Health Insurance Program) reimburse dentists for fluoride varnish treatments in children, and most state Medicaid programs reimburse medical providers for applying fluoride varnish (6).

Despite evidence for fluoride varnish effectiveness in caries prevention and its broad clinical acceptance, little systematically collected prospective data on its safety exist. The Food and Drug Administration (FDA) regulates clinical fluoride varnish use as a Class II medical device (7). After FDA’s 510(k) premarket notification process (8), fluoride varnish was cleared as a cavity liner and desensitizer, or cavity varnish (9). Using fluoride varnish for caries prevention in children or adults is considered an off-label use, because anticaries agents are considered drugs, not devices (10). The FDA advises that “good medical practice and the best interests of the patient require that physicians use legally available drugs, biologics, and devices according to their best knowledge and judgment. If physicians use a product for an indication not in the approved labeling, they have the responsibility to be well informed about the product, to base its use on firm scientific rationale and on sound medical evidence, and to maintain records of the product’s use and effects” (10).

The National Institute of Dental and Craniofacial Research’s (NIDCR’s) initiation of the Early Childhood Caries Collaborating Centers (EC4) in 2008 provided an opportunity to prospectively and systematically collect data on fluoride varnish safety. NIDCR awardees based at the University of Colorado Anschutz Medical Campus (UCAMC), University of California, San Francisco (UCSF), and Boston University (BU) each conducted a randomized controlled trial (RCT) on caries prevention in young children. Each trial used fluoride varnish in its protocol and systematically queried each child’s parents or legal guardians (hereinafter referred to as parents) about adverse events after each fluoride varnish treatment. The objective of this study was to assess the incidence of adverse events related to fluoride varnish treatment in young children in these 3 trials.

## Methods

### Study participants and data sources

We obtained data on adverse events and serious adverse events that resulted in medically attended visits in children enrolled in 3 RCTs under the auspices of the NIDCR-supported EC4. Each RCT used generally accepted definitions for identifying adverse events and serious adverse events (11). Each RCT tested an intervention designed to reduce caries incidence in young children, and each used fluoride varnish treatments. Fluoride varnish was a randomized component in 1 RCT; the other 2 RCTs assigned all enrolled children to receive fluoride varnish. A single data coordinating center at UCSF served all 3 EC4 trials, and NIDCR used a single data and safety monitoring board to oversee them.

The participating EC4 research centers (Table 1) are the Center for Native Oral Health Research at UCAMC (12); the Center to Address Disparities in Children’s Oral Health at UCSF (13); and the Northeast Center for Research to Evaluate and Eliminate Dental Disparities at BU (14). Details on each trial, including inclusion and exclusion criteria, are available at ClinicalTrials.gov (12–14). No children were excluded from any trial because of a history of asthma, peanut or nut allergies, or other food allergies.

**Table 1:** Design Elements and Participant Characteristics of 3 Randomized Controlled Trials Conducted Under the Auspices of the NIDCR-Supported Early Childhood Caries Collaborating Centers

Element or Characteristic	Location of Study Team			Total
	University of Colorado Anschutz Medical Campus^a^	University of California, San Francisco	Boston University	
Name	Preventing Caries in Preschoolers: Testing a Unique Service Delivery Model in American Indian Head Start Programs	Glass Ionomer Sealant and Fluoride Varnish Trial to Prevent Early Childhood Caries	Tooth Smart Healthy Start: Oral Health Advocates in Public Housing	—
ClinicalTrials.gov identifier	NCT01116739	NCT01129440	NCT01205971	—
Institutional review board approval	Colorado Multiple Institutional Review Board	University of California, San Francisco, Committee on Human Research	Boston University Medical Campus Institutional Review Board	—
Location	52 Head Start Centers on an American Indian reservation in the southwestern United States	2 Community health centers in San Diego County, California	26 Public housing developments in the Boston, Massachusetts, area	—
Race/ethnicity of children	American Indian	Hispanic	Hispanic, African American, non-Hispanic white	—
Dates of trial of onset and completion	September 2011–December 2014	June 2011–March 2016	January 2011–January 2017	—
Total no. of children enrolled	1,016	597	1,837	3,450
No. of children enrolled to receive fluoride varnish	518	597	1,837	2,952
Baseline eligibility age range of children, y	3–5	2.5–3	0–5	0–5
Mean baseline age of children enrolled to receive fluoride varnish, y (SD)	3.6 (0.5)	3.3 (0.5)	3.0 (1.8)	3.2 (1.5)
Frequency of fluoride varnish application	4 Times per Head Start school year	Semiannually	Quarterly	—
Maximum no. of fluoride varnish applications per protocol	4^b^	6	9	—

Abbreviations: NIDCR, National Institute of Dental and Craniofacial Research; SD, standard deviation.

a Intervention group only.

b Children who enrolled in fall 2011 (n = 55) were eligible for up to 8 fluoride varnish applications. Most children who enrolled in fall 2011 and all children enrolled in fall 2012 were eligible for 4 applications.

The UCAMC trial enrolled 1,016 children aged 3 to 5 years in Navajo Nation Head Start programs to test whether specially trained community lay health workers who administered fluoride varnish (up to 4 times per school year) and oral health promotion education (up to 5 times per school year) in Head Start classrooms for up to 2 years, reduced the number of decayed, missing due to caries, or filled primary tooth surfaces (dmfs) compared with the number of dmfs in nonintervention children; 518 children were randomized to receive fluoride varnish. In the intervention group, fluoride varnish applications were spaced 6 weeks apart on average. Each fluoride varnish application used Vanish (3M ESPE), a 5% sodium-fluoride white varnish with tri-calcium phosphate in 0.5-mL sealed-unit–dose packages, and a prepackaged brush. This RCT collected data from September 2011 to December 2014.

The UCSF trial enrolled children aged 2.5 to 3 years, primarily Hispanics, at 2 community health centers in San Diego County, California, to compare the caries prevention efficacy of fluoride varnish applied every 6 months with the efficacy of fluoride varnish applied every 6 months combined with annual fluoride-releasing glass ionomer sealants to eligible occlusal surfaces of primary molar teeth. The primary outcomes were caries incidence and caries increment at 3 annual follow-ups. Incidence refers to any increase in dmfs (ie, a binary indicator of Δ_dmfs_ > 0), whereas increment refers to a (positive) change in dmfs count over time (ie, Δ_dmfs_). Fluoride varnish was applied semiannually to 597 participants from baseline through the 30-month visit. Each fluoride varnish application used a 0.25-mL–unit dose of CavityShield (3M ESPE), a 5% sodium-fluoride varnish, per mouth and a prepackaged brush. This RCT collected data from June 2011 to March 2016.

The BU trial enrolled children aged 0 to 5 years, residing in public housing developments in the greater Boston area. A total of 1,837 children were enrolled in 26 housing developments. All children were assigned to receive fluoride varnish. The RCT compared 2 community-based multimodal interventions: 1) motivational interviewing (counseling) by dental health advocates for participating parents, child fluoride varnish application, child oral health assessment, and referral to dental health services; or 2) written oral health education materials on the prevention of early childhood caries, child fluoride varnish application, child oral health assessment, and referral to dental health services. All intervention components, including fluoride varnish, were delivered quarterly for 2 years. The primary outcome was 2-year incidence of early childhood caries. Each fluoride varnish application used a 0.40-mL–unit dose of CavityShield (3M ESPE) 5% sodium-fluoride varnish and a prepackaged brush. This RCT collected data from January 2011 to January 2017.

### Determination of adverse events after each fluoride varnish application

Each RCT protocol specified a procedure for contacting each child’s parent after each fluoride varnish application. Typically, a study staff member made contact by telephone and used a standardized script. The purpose of the contact was to assess whether the child experienced any adverse health events after his or her most recent study-related fluoride varnish application, the nature of any such event, and whether the event resulted in a medically attended visit. To assess safety of fluoride varnish treatments, study-related adverse events were defined as health events that resulted in a medically attended visit within a prescribed timeframe (termed the fluoride varnish–adverse event [FV–AE] window) after the fluoride varnish application. The UCSF and BU trials targeted adverse events that occurred within the first 7 calendar days after fluoride varnish application. The UCAMC trial targeted adverse events that occurred 3 to 10 business days after fluoride varnish application. 

In addition to the data collected as part of active safety monitoring after each fluoride varnish application during the FV–AE windows, we also recorded other health-related information that parents reported in telephone calls or in person outside the FV–AE windows. The UCSF and BU trials used medical history forms that asked parents the following yes/no questions about their study-enrolled child: Does your child have an allergy to fluoride varnish or any of its components? Has your child ever had any complications with dental treatment? Both questions also had corresponding free-text fields where details could be provided. The BU trial asked these questions every 3 months, and the UCSF trial, every 6 months. The UCAMC trial used an “extemporaneous encounter form.” It was completed by study staff members if they had a chance encounter with a study participant in which the participant revealed health-related information about a study-enrolled child. We used data collected by each trial using such forms to determine whether any additional fluoride varnish–related adverse events were reported. 

Each adverse event identified by study staff members was subsequently adjudicated by a clinician principal investigator to determine whether it was related to participation in the RCT. The NIDCR medical monitor, the data and safety monitoring board, and each trial’s institutional review board reviewed all adverse events at least annually. The principal investigators reported serious adverse events within 72 hours of learning of an event to NIDCR’s clinical research operations management systems contractor. Also, as appropriate, the RCT reported relevant occurrences to FDA by using the agency’s voluntary Safety Information and Adverse Event Reporting Program’s Form 3500 (15).

### Data analysis

We generated the following summary statistics for each RCT: number of enrolled children who had at least 1 fluoride varnish application, total number of completed fluoride varnish applications, mean number of fluoride varnish applications per child, and the distribution of the number of fluoride varnish applications per child. We also pooled these data for all 3 RCTs. We calculated the timing of the intervals among repeated fluoride varnish applications in each trial and tabulated data on follow-up contacts with parents. Lastly, we calculated the number of adverse events reported for each RCT and across all RCTs. Because we found no trial-related adverse events or serious adverse events after fluoride varnish treatment, we estimated the 1-sided 95% confidence interval (CI) upper bound for adverse events or serious adverse events by using the rule-of-three approximation (16). The calculation also required adjustment for intracluster correlation (ICC) from the clustered sampling designs used in the UCAMC and BU trials as well as potential intraperson correlation of adverse events across repeated fluoride varnish applications. However, because we found no trial-related adverse events, ICC values could not be estimated from the data. Instead, we estimated 95% CI upper bounds for 3 ICC values (0, 0.5, and 1.0) representing levels of intraperson correlation of repeated response. For this calculation, cluster size was specified as the per-child average number of fluoride varnish applications among children with at least 1 fluoride varnish application.

## Results

Across the 3 trials, 2,424 children received 10,249 fluoride varnish applications (Table 2). The number of completed fluoride varnish applications in each trial partly depended on the scheduled fluoride varnish application frequency and trial duration, which varied by trial (Table 1). Of 2,424 children with at least 1 fluoride varnish application, 1,863 (76.9%) had at least 3 applications and 1,562 (64.4%) had at least 4 applications. The mean intervals between fluoride varnish applications varied across trials, from 39 to 182 days. On average, across trials, each treated child received 4.2 fluoride varnish applications (Table 2).

**Table 2:** Descriptive Statistics on Fluoride Varnish Applications in 3 Randomized Controlled Trials Conducted Under the Auspices of the NIDCR-Supported Early Childhood Caries Collaborating Centers

Characteristic	Location of Study Team			Total
	University of Colorado Anschutz Medical Campus^a^	University of California, San Francisco^b^	Boston University^c^	
**No. of children with 0 fluoride varnish applications**	39	1	488	528
**No. of children with ≥1 fluoride varnish application**	479	596	1,349	2,424
**Total no. of fluoride varnish applications completed**	1,893	3,188	5,168	10,249
**Mean no. of fluoride varnish applications per child^d^**	4.0	5.3	3.8	4.2
**Mean (SD) no. of days between fluoride varnish treatments**	39.1 (8.8)	181.5 (20.7)	96.7 (32.4)	NA
**Median (IQR) no. of days between fluoride varnish treatments**	36 (34–46)	182 (175–188)	95 (77–115)	NA
**Range of no. of days between fluoride varnish treatments**	14–70	83–532	16–595	NA
**No. (%) of fluoride varnish applications per child**				
1	21 (4.1)	26 (4.4)	220 (16.3)	267 (11.0)
2	27 (5.2)	27 (4.5)	240 (17.8)	294 (12.1)
3	77 (14.9)	22 (3.7)	202 (15.0)	301 (12.4)
4	304 (58.7)	21 (3.5)	208 (15.4)	533 (22.0)
5	3 (0.6)	42 (7.0)	159 (11.8)	204 (8.4)
6	4 (0.8)	458 (76.8)	134 (9.9)	596 (24.6)
7	12 (2.3)	NA^e^	82 (6.1)	94 (3.9)
8	31 (6.0)	NA^e^	79 (5.9)	110 (4.5)
9	NA^e^	NA^e^	25 (1.9)	25 (1.0)

Abbreviations: IQR, interquartile range; NA, not applicable; NIDCR, National Institute of Dental and Craniofacial Research; SD, standard deviation.

a Intervention group only. Children who enrolled in fall 2011 (n = 55) were eligible for up to 8 fluoride varnish applications. Most children who enrolled in fall 2011 and all children enrolled in fall 2012 were eligible for 4 applications. Fluoride varnish applied 4 times per Head Start school year.

b Fluoride varnish applied semiannually.

c Fluoride varnish applied quarterly.

d Of those with ≥1 fluoride varnish application.

e Per protocol, this number of fluoride varnish applications was not planned.

The 3 trials reached from 75.7% (BU) to 96.9% (UCSF) of parents at follow-up (Table 3). For the trials at BU and UCSF, the number of contact attempts totaled 13,769 telephone calls, corresponding to approximate average of 1.6 contact attempts per successful contact (Table 3). Across all 3 trials, the 8,548 successful contacts identified 8 adverse events and 1 serious adverse event, each from a different study participant (Table 3).

**Table 3:** Fluoride Varnish–Related Adverse Events Among Children With at Least 1 Fluoride Varnish Application, 3 Randomized Controlled Trials Conducted Under the Auspices of the NIDCR-Supported Early Childhood Caries Collaborating Centers

Variable	Location of Study Team			Total
	University of Colorado Anschutz Medical Campus^a^	University of California, San Francisco^b^	Boston University^c^	
**No. of fluoride varnish applications completed**	1,893	3,188	5,168	10,249
**Follow-up on adverse events**				
No. of contact attempts	NA^d^	4,612	9,157	13,769
No. (%) of successful contacts^e^	1,546 (81.7)	3,090 (96.9)	3,912 (75.7)	8,548 (83.4)
Average no. of attempts per contact	NA^d^	1.5	2.3	1.6
**Adverse events or serious adverse events that resulted in a medically attended visit**				
No. (%) of all-cause adverse events or serious adverse events^f^	8 (0.09)	1 (0.03)	0	9 (0.11)
No. of study-related adverse events or serious adverse events	0	0	0	0

Abbreviations: NA, not applicable NIDCR, National Institute of Dental and Craniofacial Research.

a Intervention group only. Children who enrolled in fall 2011 (n = 55) were eligible for up to 8 fluoride varnish applications. Most children who enrolled in fall 2011 and all children enrolled in fall 2012 were eligible for 4 applications. Fluoride varnish applied 4 times per Head Start school year.

b Fluoride varnish applied semiannually.

c Fluoride varnish applied quarterly.

d Data not electronically recorded.

e Percentage calculated by using the corresponding number of fluoride varnish applications completed as the denominator.

f Percentage calculated by using the corresponding number of successful contacts as the denominator.

The 8 all-cause adverse events consisted of reports of a cold, cold or influenza, cough or fever, influenza or ear infection, fever, pneumonia, streptococcus infection, or viral infection. The single all-cause serious adverse event was frostbite. The principal investigators determined that all 9 events were not related to the trial or to fluoride varnish treatment. Subsequent external oversight review concurred with the determinations.

Using the rule-of-three approximation, we found upper-bound estimates for the expected percentage of trial-related adverse events and serious adverse events to be 0.035% (ICC = 0), 0.092% (ICC = 0.5), and 0.148% (ICC = 1.0). Thus, the upper-bound estimates ranged from one-twenty-ninth of 1% (ICC = 0) to one-seventh of 1% (ICC = 1).

### Other reported incidents

After the FV–AE window had closed, in 10 children, across 2 studies, parents reported that their children were allergic to fluoride varnish. However, 6 of those 10 parents reported at a subsequent study visit that their child was not allergic to fluoride varnish. In 1 such case, a parent reported that her child had blisters on her tongue and difficulty swallowing after the 2 most recent fluoride varnish applications, even though the report was made 6 months after the most recent fluoride varnish application and no problems had been reported by the parent during follow-up calls that occurred within 1 week of each fluoride varnish application. Although the case did not meet the trial protocol’s definition of an adverse event, after consulting the trial’s NIDCR medical monitor, the trial’s principal investigator reported this case to the FDA via Form 3500. In another 2 children, the following incidents were reported by parents after the FV–AE window had closed: 1 child had a case of herpetic lesions that was medically attended, but the incident occurred 3 days after the trial’s FV–AE window had closed and was determined to be unrelated to fluoride varnish; and another child vomited while receiving a fluoride varnish application. Finally, in 2 other children, the following incidents occurred within a trial’s follow-up FV–AE window, but neither incident was medically attended: 1 parent reported that a child had diarrhea, and another parent reported that a child had “a rash under (his/her) lip less than dime size” after the first of 8 total fluoride varnish applications and “a little rash around (his/her) mouth” after the seventh application.

## Discussion

In 1995, fluoride varnish received FDA clearance as a Class II medical device in the United States. Fluoride varnish has been used in Europe for caries prevention in children and adults since 1964 (17). Over several decades, evidence has mounted on both its clinical effectiveness and safety (2,3,17–19). Despite widespread use of fluoride varnish, evidence on fluoride varnish–related adverse events is minimal and few large-scale fluoride varnish clinical trials have been conducted that include a data and safety monitoring board and a standardized protocol for soliciting, monitoring, documenting, and following up on adverse events.

Potential concerns about the safety of fluoride varnish relate primarily to systemic effects from chronic fluoride ingestion, including increased risk of enamel fluorosis and renal toxicity, with acute topical effects predominantly related to contact hypersensitivity involving the oral mucosa. Previous reviews did not report evidence of acute toxicity from fluoride varnish application (17,19). A Cochrane systematic review in 2013 (18) found “little information concerning possible adverse effects” of fluoride varnish treatment.

Fluoride varnish use outside of dental settings has increased in the United States. In the North Carolina program, “Into the Mouths of Babes,” primary medical care providers treated more than 250,000 children aged 0 to 3 years with no reports of fluoride varnish–related adverse events (20,21). However, the estimates generated by that study may have underrepresented the incidence of adverse events because the estimates depended on providers taking the time to report them.

Fluorosis (ie, changes in the appearance of tooth enamel that are caused by long-term ingestion of fluoride during the time teeth are forming) is cited as a potential concern, although there is no published evidence indicating professionally applied fluoride varnish is a risk factor for fluorosis from prolonged or repeated exposure even in children under 6 years old (19, 22). Fluorosis is unlikely if not impossible to occur with the recommended frequency and dosage schedules for fluoride varnish. After fluoride varnish application, plasma fluoride concentrations peak within 2 hours and then rapidly decrease (23). The plasma fluoride concentrations reached and the kinetics were similar to those found after brushing with fluoridated toothpaste (24). A study of the pharmacokinetics of fluoride varnish application 5 hours after application in children aged 12 to 15 months showed systemic exposure well below levels associated with acute toxicity and similar to control levels (25). The USPSTF also found no evidence of “risk for fluorosis with fluoride varnish application” (3). Other potential health effects of fluoride ingestion include gastric irritation and nausea. Compared with ingestion of fluoride from other forms of fluoride, however, ingestion from fluoride varnish is less because only a sparse amount is required for treatment (23).

The FDA Manufacturer and User Facility Device Experience Database (26) reported 2 cases of severe anaphylactic or allergic reactions to fluoride varnish treatment. However, these incidents were associated with 1 fluoride varnish product (Recaldent [Recaldent Pty Ltd]), which also contains casein phosphopeptides, which are derived from the casein protein in milk, in combination with amorphous calcium phosphate. Reactions reported were likely related to the antigenic properties of casein phosphopeptide–amorphous calcium phosphate and not fluoride varnish itself.

Contact irritation of the oral mucosa resulting from fluoride varnish has been sporadically reported (19). Such reactions likely relate to the colophony base serving as fluoride’s vehicle and are described as mucosal rashes, aphthous ulcer-like lesions, or a short-term “burning sensation” (19,27). The manufacturers’ product information typically notes that fluoride varnish is contraindicated in patients with ulcerative gingivitis or stomatitis or patients with known sensitivity to colophony or other ingredients. Colophony is a complex mixture of more than 100 compounds from pine trees, and varies with climate, type of pine tree, and extraction and storage methods (28). The main colophony component is resin and/or rosin acid derived from the tree wood and not from tree nuts. Manufacturers thus do not name tree nut allergies as contraindications. Meaningful conclusions about the prevalence of colophony allergy are difficult because of colophony’s widespread use outside of health care settings, including in paper, ink, chewing gum, adhesives, and detergents (28).

Among 376 children followed up to 2 years in an RCT (29), no adverse events were reported in association with fluoride varnish applications, including enrolled children with asthma diagnoses (29). In contrast, a single case report in 1998 made note of a fluoride varnish adverse event in an asthmatic child (30). More recently, in a 2-year RCT in Brazil (31), among 200 children treated with fluoride varnish or placebo varnish, investigators reported only a single incident of “burning sensation” in a child receiving placebo varnish and no adverse events in any children with asthma. Fluoride bioavailability studies after fluoride varnish application found urinary fluoride temporarily increased and returned to baseline within 24 hours, concluding fluoride varnish is safe for children (32,33).

The data used in our analysis had some limitations. First, slightly different FV–AE windows were used among the 3 trials (7 calendar days by the UCSF and BU trials; 3 to 10 business days by the UCAMC trial); however, any acute adverse events would have been reported in either window. Another limitation was that the UCAMC trial did not record the number of telephone attempts required to reach parents; nevertheless, that trial successfully completed more than 80% of the telephone calls made during the FV–AE window.

Among the 3 RCTs examined in our study, we found no evidence of fluoride varnish–related adverse events after more than 10,000 fluoride varnish applications in more than 2,400 children. Our study is the first large-scale systematic prospective assessment of fluoride varnish–related adverse events in young children. The use of fluoride varnish for caries prevention in young children is expected to increase as a result of the USPSTF recommendation (3) and growth in government and commercial insurance coverage for physicians and dentists who apply fluoride varnish. Safety concerns are likely to remain an important consideration in decision making for health care providers and families of young children. Thus, our study is a timely contribution to the evidence base. Future clinical studies of fluoride varnish should systematically assess data on safety and investigate the effectiveness of fluoride varnish in preventing caries in the primary dentition, including the optimal dose or frequency of fluoride varnish treatments (34,35).

## Post-Test Information

To obtain credit, you should first read the journal article. After reading the article, you should be able to answer the following, related, multiple-choice questions. To complete the questions (with a minimum 75% passing score) and earn continuing medical education (CME) credit, please go to http://www.medscape.org/journal/pcd. Credit cannot be obtained for tests completed on paper, although you may use the worksheet below to keep a record of your answers. You must be a registered user on Medscape.org. If you are not registered on Medscape.org, please click on the "Register" link on the right hand side of the website to register. Only one answer is correct for each question. Once you successfully answer all post-test questions you will be able to view and/or print your certificate. For questions regarding the content of this activity, contact the accredited provider, CME@medscape.net. For technical assistance, contact CME@webmd.net. American Medical Association’s Physician’s Recognition Award (AMA PRA) credits are accepted in the US as evidence of participation in CME activities. For further information on this award, please refer to http://www.ama-assn.org/ama/pub/about-ama/awards/ama-physicians-recognition-award.page. The AMA has determined that physicians not licensed in the US who participate in this CME activity are eligible for ***AMA PRA Category 1 Credits™***. Through agreements that the AMA has made with agencies in some countries, AMA PRA credit may be acceptable as evidence of participation in CME activities. If you are not licensed in the US, please complete the questions online, print the AMA PRA CME credit certificate and present it to your national medical association for review.

## Post-Test Questions

### Study Title: Absence of Fluoride Varnish–Related Adverse Events in Caries Prevention Trials in Young Children, United States

#### CME Questions

1. Your patient is a 2-year-old boy in whom fluoride varnish is being considered for caries prevention. According to the prospective systematic assessment by Garcia and colleagues, which of the following statements about findings regarding adverse events (AEs) of fluoride varnish in young children enrolled in caries prevention trials is ***most*** accurate?
  - Because each child received only 1 to 2 treatments, it is difficult to confirm the safety of fluoride varnish
  - Because of the small sample size, it is difficult to confirm the safety of fluoride varnish
  - Rate of fluoride varnish-related AEs was 1.0%
  - A total of 8 all-cause AEs (cold, cold/flu, cough/fever, flu/ear infection, fever, pneumonia, streptococcus or viral infection) and 1 serious AE (frostbite) were all unrelated to fluoride varnish treatment
2. According to the prospective systematic assessment by Garcia and colleagues, which of the following statements about the clinical implications of these findings regarding AEs of fluoride varnish in young children enrolled in caries prevention trials is ***correct***?
  - Findings of this study do not support the safety of fluoride varnish as an effective prevention intervention for caries in young children
  - Fluoride varnish received clearance from the US Food and Drug Administration as a class II medical device in the United States in 1995
  - Fluoride varnish has only been in widespread use for the past 2 decades
  - Clinical guidelines have not recommended routine use of fluoride varnish
3. According to the prospective systematic assessment by Garcia and colleagues, what is a potential concern regarding the safety of fluoride varnish?
  - Systemic effects from long-term ingestion of fluoride may include increased risks for enamel fluorosis and renal toxicity
  - Acute topical AEs predominantly involve enamel discoloration
  - At recommended frequency and dosage schedules for fluoride varnish, fluorosis is likely to affect 1% to 2% of children younger than 6 years
  - Severe anaphylactic or allergic reactions have been reported with a variety of fluoride varnish products

**Table:** Evaluation

**1. The activity supported the learning objectives.**				
**Strongly Disagree**	** **	** **	** **	**Strongly Agree**
1	2	3	4	5
**2. The material was organized clearly for learning to occur.**				
**Strongly Disagree**	** **	** **	** **	**Strongly Agree**
1	2	3	4	5
**3. The content learned from this activity will impact my practice.**				
**Strongly Disagree**	** **	** **	** **	**Strongly Agree**
1	2	3	4	5
**4. The activity was presented objectively and free of commercial bias.**				
**Strongly Disagree**	** **	** **	** **	**Strongly Agree**
1	2	3	4	5
